# Cognitive profile in patients with a first-ever lacunar infarct with and without silent lacunes: a comparative study

**DOI:** 10.1186/1471-2377-13-203

**Published:** 2013-12-16

**Authors:** Lorena Blanco-Rojas, Adrià Arboix, David Canovas, Marta Grau-Olivares, Joan Carles Oliva Morera, Olga Parra

**Affiliations:** 1 Cerebrovascular Division, Department of Neurology, Capio-Hospital Universitari del Sagrat Cor, Universitat de Barcelona, C/ Viladomat 288, E-08029, Barcelona, Catalonia, Spain; 2 CIBER de Enfermedades Respiratorias (CB06/06), Instituto Carlos III, Madrid, Spain; 3 Department of Neurology, Hospital Parc Taulí, Sabadell, Barcelona, Catalonia, Spain; 4 Fundació Parc Taulí, Corporació Sanitària Universitària Parc Taulí, Universitat Autònoma de Barcelona, Sabadell, Barcelona, Catalonia, Spain; 5 Universitat Autònoma de Barcelona, Cerdanyola del Vallès, Barcelona, Catalonia, Spain; 6 Department of Pneumology, Capio-Hospital Universitari del Sagrat Cor, Universitat de Barcelona, Barcelona, Catalonia, Spain

**Keywords:** California verbal learning test, Lacunar infarction, Neuropsychological abnormalities, MRI, Semantic fluency, Silent multiple lacunar infarctions, Vascular cognitive impairment

## Abstract

**Background:**

The detection of early neuropsychological abnormalities as precursors of cognitive decline of vascular origin in patients with lacunar stroke is a subject of increasing interest. The objective of this study was to assess whether there were differences in the performance of a battery of neuropsychological tests in first-ever lacunar stroke patients with and without associated silent multiple lacunar infarctions found incidentally on the brain magnetic resonance imaging (MRI) scan.

**Methods:**

A total of 72 consecutive patients with first-ever lacunar infarction were studied 1 month after stroke. All patients underwent a comprehensive neuropsychological evaluation, which included the California Verbal Learning Test (CVLT), Phonetic Verbal Fluency Test (PMR), Semantic Verbal Fluency Test (category “animals”), Digit Span Forward and Backward from the Wechsler Adult Intelligence Scale (WAIS-III), and Mini-Mental State Examination (MMSE).

**Results:**

A total of 38 patients (52.7%) had silent multiple lacunar infarcts, with corona radiata as the most frequent topography (*P* < 0.023). White matter hyperintensities (leukoaraiosis) were observed in 81.1% of patients with silent multiple lacunar infarcts and in 50% with a single lacunar infarction (*P* < 0.007). Patients in both groups showed similar scores in the MMSE, but those with associated silent lacunar infarctions showed a poorer performance in the semantic fluency test (*P* < 0.008) and in short delayed verbal memory (*P* < 0.001). In both cases, however, leukoaraiosis was not statistically significant in multivariate linear regression models adjusted by confounding covariates. In these models, multiple silent lacunar infarctions and education were independent predictors of poor performance in the semantic fluency test and in short delayed verbal memory.

**Conclusions:**

The presence of silent multiple lacunar infarctions documented on brain MRI scans in patients with first-ever lacunar stroke was associated with mild neuropsychological abnormalities, particularly in the performance of executive functions (semantic fluency) and short delayed verbal memory. According to these findings, in the initial stages of small vessel disease, mild neuropsychological abnormalities appear to be related to lacunes rather than to leukoaraiosis or perivascular hyperintensities of vascular cause.

## Background

Lacunar infarcts (LI) or lacunes account for 20-25% of all ischemic strokes [1,2]. According to recent studies, to distinct entities in patients with first-ever LI can be distinguished: 1) patients with a single LI in whom well known cardiovascular risk factors are present and 2) patients with multiple LI, in whom a high frequency of hypertension and leukoaraiosis have been found [3,4]. Also, mild neuropsychological abnormalities mainly executive dysfunction have been reported in 57% of patients with acute lacunar stroke [5] especially in patients with pure motor hemiparesis and atypical lacunar syndromes. In these circumstances, although general cognitive performance is usually normal, 55% of cases fulfill criteria of mild cognitive impairment of vascular type [6]. Identification of early stages of subcortical vascular dementia is an important area of research because this form of vascular dementia is one of the most common causes of cognitive decline in the elderly population [7,8].

The potential impact of silent multiple lacunar infarctions on neuropsychological function in patients with clinically documented LI remains unclear. Therefore, the objective of this study was to assess whether there were differences in the performance of a battery of neuropsychological tests in first-ever lacunar stroke patients with and without associated silent multiple lacunar infarctions found incidentally on the brain magnetic resonance imaging (MRI) scan.

## Methods

### Patients

The study population consisted of 72 consecutive patients with first-ever LI admitted to the Department of Neurology of the Capio-Hospital Universitari Sagrat Cor, in Barcelona, Spain, between January 2006 and December 2011. All patients presented a lacunar syndrome according to the Miller-Fisher’s classification [1]. Patients with cortical and/or subcortical non-lacunar infarct or intracerebral hemorrhage documented by MRI studies were excluded from the study as were those with severe cardiovascular disease, renal insufficiency, liver dysfunction, neoplastic or chronic disease, major depression and other psychiatric comorbidity (DSM-IV-R). Patients with impaired cognitive performance (Mini-Mental State Examination [MMSE] score < 24) were also excluded.

The definitions of cerebrovascular risk factors and classic lacunar syndromes, including pure motor stroke, pure sensory stroke, sensorimotor stroke, ataxic hemiparesis, dysarthria-clumsy hand and atypical lacunar syndromes (patients presenting isolated dysarthria, dysarthria with facial paresis or isolated hemiataxia) were those used in previous studies [5,6,9]. Prior to conducting the study, approval was obtained from Ethical Committee on Clinical Research of the hospital. Written informed consent was obtained from all patients.

### MRI examination

MRI studies were performed using a General Electric 1.5 Tesla Sigma system (General Electric, Milwaukee, WI) and the following sequences: T1-weighted (TR = 479 ms, TE = 13 ms, FOV = 270/1.1, slice thickness 5.0 mm, GAP 1.0); T2-weighted (TR = 4885 ms, TE = 120 ms, FOV = 220/1.1, slice thickness 5.0 mm, GAP 2.0); fluid-attenuated inversion recovery (FLAIR) (TR = 8000 ms, TE = 120 ms, FOV = 240/1.1, slice thickness 5.0 mm, GAP 1.2); protonic density (TR = 3400 ms, TE = 17 ms, FOV = 240/1.1, slice thickness 5.0 mm, GAP 1.0); and diffusion sequences (TR = 3350 ms, TE = 74 ms, FOV = 250/1.1, slice thickness 5.0 mm, GAP 1.0). Two senior radiologists determined the presence and topography of acute and chronic, single and/or multiple silent lacunar infarcts by visual inspection of the T1, FLAIR, and T2 sequences of MRI scans. Patients were divided into two groups, those with a single LI and those with a single LI in association with multiple silent lacunar infarcts. Also, white matter hyperintensities (leukoaraiosis) were evaluated from T2-weighted, FLAIR and diffusion sequences and classified as present versus absent.

### Neuropsychological studies

A battery of neuropsychological tests [10] was administered to all patients 1 month after the index admission. These included the California Verbal Learning Test (CVLT), Phonetic Verbal Fluency Test (PMR), Semantic Verbal Fluency Test (category “animals”), and Digit Span Forward and Backward from Wechsler Adult Intelligence Scale (WAIS-III). The Spanish versions of these instruments were used [11].

The CVLT is a standardized test for verbal learning and memory function developed to assess both the amount of material learned, recalled, and recognized, as well as qualitative aspects of how the verbal learning occurs or fails. A list of 16 words (List A) is presented five times. The words are equally drawn from four semantic categories with no consecutive words from the same category. Immediately after the fifth trial, a new list is read to the participant (List B) and asked to recall it. A short delayed recall test is presented immediately after recall of List B, where the participant is asked to recall the words in List A. A long delayed recall test (CVLT-Long Delayed) is presented after a further 20 minutes interval. Finally, a “yes-no” recognition test consisting in the 16 items of List A, eight from List B and 20 random distracter items is presented [12]. The PMR test is used to assess semantic knowledge, retrieval ability and executive functioning. The patients have to say words beginning with letter “p” “m” and ”r” in 60 seconds. In the Semantic Verbal Fluency Test (category “animals”), participants had to say as many words as possible from the semantic category “animals” in 60 seconds. The final measure is the total number of words. The Digit Span Forward and Backward Test is a subtest from WAIS-III, which is used to explore the short delayed verbal memory, working memory, and attention ability. The Digit Span Forward consists of serial numbers that the patient has to repeat in the same order than the evaluator. In the Backwards Test the patient has to say the serial numbers in the other way round.

### Statistical analysis

Differences in demographics, vascular risk factors, clinical features, and results of neuropsychological tests between patients with single LI and those with associated multiple silent LIs were analyzed with the Student’s *t* test for continuous variables and the chi-square (*χ*^2^) test or the Fisher’s exact probability test (when appropriate) for categorical data. The degree of association of individual variables with multiple silent LIs was estimated by the odds ratio (OR) and the 95% confidence interval (CI). Multiple linear regression analysis was performed to assess the effect of leukoaraiosis on neuropsychological performance adjusted by confounding covariates. Statistical significance was set at *P < 0.05*.

## Results

There were 36 men and 36 women, with a mean (standard deviation, SD) age of 75.4 (9.2) years. Thirty-four (47.2%) patients presented a single LI and in the remaining 38 (52.8%), associated silent multiple LIs were detected on the MRI scan. Table 1 shows the comparison of demographic variables, vascular risk factors, clinical features, and infarct topography between the groups of single and multiple silent LIs. Statistically significant differences (*P* = 0.014) were only found in LI topography, with higher percentages of multiple silent LIs in the basal ganglia and corona radiata, and the presence of leukoaraiosis, which was significantly more frequent in patients with multiple silent LIs (*P* = 0.007). The remaining variables including clinical types of LI were similarly distributed in the two study groups. The mean (SD) MMSE score was 27.8 (2.3) in the group of single LI and 28 (2) in the group of multiple silent LIs (*P* = 0.686).

**Table 1:** Demographic, clinical and cognitive features of patients with single lacunar infarction (LI) and patients with associated multiple silent LIs

**Variables**	**Lacunar stroke patients**		**Odds ratio (95% CI)**	***P *** **value**	t^*^
	**Single LI**	**Multiple silent**			
	**(n = 34)**	**LIs (n = 38)**			
Age, years, mean (SD)	73.1 (11.3)	77.3 (7.7)		0.999	-1.834
Women, no. (%)	17 (50)	19 (50)		0.999	0.000
Education, mean (SD)^†^	8.9 (3.7)	8.4 (2.9)		0.71	0.672
MMSE, score, mean (SD)	27.8 (2.3)	28 (2.0)		0.686	-0.406
High BP >140/90 mm Hg, no. (%)	23 (67.6)	29 (76.3)	1.541 (0.546–4.347)	0.414	-0.812
Diabetes mellitus, no. (%)	10 (29.4)	13 (34.2)	1.248 (0.461–3.381)	0.663	-0.430
Dyslipidemia, no, (%)	10 (29.4)	12 (31.5%)	1.108 (0.405–3.029)	0.842	-0.170
Toxic habits, no. (%)^‡^	14 (41.2%)	15 (39.5%)	1.073 (0.418–2.756)	0.883	-0.372
Lacunar syndrome, no. (%)				0.381	0.360
Pure motor hemiparesis	9 (26.5)	10 (26.3)	-		
Pure sensory syndrome	8 (23.5)	10 (26.3)	1.125 (0.308–4.104)		
Dysarthria–clumsy hand	5 (14.7)	9 (23.7)	1.62 (0.392–6.678)		
Sensorimotor syndrome	4 (11.8)	1 (2.6)	0.225 (0.021–2.404)		
Ataxic hemiparesis	2 (5.9)	0			
Atypical lacunar syndrome	6 (17.6)	8 (21.1)	1.2 (0.298–4.816)		
Infarct lateralization, no. (%)				0.153	-0.538
Right	15 (44.2)	17 (44.7)	-		
Left	18 (52.9)	15 (39.5)	0.735 (0.277–1.950)		
Bilateral	1 (2.9)	6 (15.8)	5.294 (0.570–49.136)		
Infarct topography, no (%)				0.014	-0.883
Basal ganglia	2 (5.9)	6 (15.8)	7.500 (1.039–54.118)		
Corona radiata	6 (17.6%)	17 (44.7%)	7.083 (1.601–31.331)		
Internal capsule	10 (29.4%)	4 (10.5%)	-		
Thalamus	9 (26.5%)	9 (23.7)	2.500 (0.567–11.011)		
Pons	7 (20.6%)	2 (5.3)	0.714 (0.101–5.035)		
Leukoaraiosis, no. (%)	17 (50)	30 (81.1)	4.286 (1.481–12.4)	0.007	-2.886

^*^t: test statistics; ^†^years of formal education; ^‡^smoking/alcohol consumption.

*Abbreviations*: *CI* confidence interval, *MMSE* Mini-Mental State Examination, *BP* blood pressure.

The results of neuropsychological tests are shown in Table 2. The group of patients with multiple silent LIs showed a poorer performance in semantic fluency (mean difference 3.265, 95% CI 0.891-5.638; *P* = 0.008) and short delayed verbal memory (mean difference 1.265, 95% CI 0.514-2.015; *P* = 0.001) as compared with the group of patients with single LI. Differences in the results of other tests were not statistically significant. There were no differences in the performance of neurological tests between the different lacunar subtypes.

**Table 2:** Results of neuropsychological tests in patients with single lacunar infarction (LI) and patients with associated multiple silent LIs

**Variables**	**Lacunar stroke patients**		**Mean difference (95% CI)**	***P *** **value**	t^*^
	**Single LI**	**Multiple silent**	** **		
	**(n = 34)**	**LIs (n = 38)**			
Phonetic verbal fluency test (PMR)	25.30 (14.90)	20.30 (10.57)	4.997 (-1.282–11.277)	0.111	1.617
Semantic fluency (animals)	14.84 (5.15)	11.84 (4.72)	3.265 (0.891–5.638)	0.008	2.746
California verbal learning test (CVLT)					
CVLT short–delay (trial 1)	4.51 (1.69)	3.25 (1.42)	1.265 (0.514–2.015)	0.001	3.366
CVLT learning (trial 1–5)	4.66 (2.74)	5.5 (2.138)	-0.833 (-2.066–0.399)	0.182	-1.349
CVLT long delay cued recall	5.96 (3.05)	5.08 (3.25)	0.896 (-0.634–2.407)	0.249	1.163
CVLT long delay recognition	12.06 (3.14)	11.69 (3.24)	0.376 (-1.170–1.902)	0.636	0.476
CVLT total learning (sum trial 1–5)	36 (10.57)	32.63 (9.5)	3.361 (-1.470–8.192)	0.170	1.389
WAIS–III digit span forward					
Direct scores	11.63 (4.24)	11.44 (2.78)	0.191 (-1.555–1.939)	0.823	0.224
Scaled scores	11.12 (3.37)	11.88 (2.61)	-0.767 (-2.210–0.675)	0.292	-1.062

^*^t: test statistics; CI: confidence interval.

Data expressed as mean (standard deviation, SD) unless otherwise stated.

In the multiple linear regression analysis to assess independent variables associated with performance of semantic fluency and short delayed verbal memory, the presence of leukoaraiosis was not statistically significant. In both models, multiple silent LIs and education were the only statistically significant variables (Table 3). Age almost reached statistical significance (*P* = 0.062) in the semantic fluency model.

**Table 3:** Variables related to performance of semantic fluency and short delayed verbal memory in the multiple linear regression analysis

**Variable**	**Coefficient (ß)**	**Standard error**	**t-statistic**	***P *** **value**
Semantic fluency model^*^				
Constant	19.845	4.825	4.112	0.000
Multiple silent lacunar infarcts	-2.255	1.027	-2.194	0.031
Leukoaraiosis	-1.312	1.180	-1.112	0.270
Age	-0.109	0.057	-1.896	0.062
Education	0.677	0.166	4.073	0.0001
Short delay verbal memory model^†^				
Constant	6.039	1.619	3.729	0.0004
Multiple silent lacunar infarcts	-0.996	0.344	-2.889	0.005
Leukoaraiosis	-0.351	0.395	-0.887	0.378
Age	-0.028	0.019	-1.453	0.151
Education	0.195	0.056	3.500	0.0008

^*^Adjusted R-squared: 0.3903; *P* value: 2.802.

^†^Adjusted R-squared: 0.3463; P value: 2.365.

## Discussion

The clinical relevance of symptomatic lacunar stroke is well established but little is known of the impact of silent lacunes incidentally found on MRI scans on cognitive function. Previous studies have shown that a first single LI may cause cognitive decline as suggested by impairment in neuropsychological performance [3-5]. The present results indicate that lacunar stroke patients with associated multiple silent LIs had significantly poorer performance in some neuropsychological tests such as semantic verbal fluency and short term memory. These findings are even more interesting considering that patients were evaluated shortly after the acute cerebrovascular event (1 month).

Our results contribute to justify the presence of two distinct phenotypic entities (in risk factors, neuroimaging and neuropsychological profile) in lacunar strokes: LI caused by one isolated symptomatic lacune and lacunar stroke caused by one symptomatic LI in association with multiple clinically silent lacunar infarctions [1-4]. Also, leukoaraiosis was significantly more frequent in patients with multiple silent LIs, although a statistically significant role as an independent predictor of performance of semantic verbal fluency and short term memory was not observed in the multivariate linear regression analysis. The underlying small vessel vasculopathy may be different in the two LI entities [3,13,14]. A diffuse arteriopathy of perforating arteries with hyaline deposition (lipohyalinosis) in the group of multiple silent LIs and localized small vessel microatheroma at the origin of the deep perforating arteries (in single LI group). Although speculative, distinguishing these two clinical LI entities may enable more appropriate therapy.

In the present study, the neuropsychological impairment in the group of patients with associated multiple silent LIs probably results from the interruption of prefrontal-subcortical loops by LIs and white matter lesions. Interruptions of dorsolateral prefrontal-subcortical loop results in executive dysfunction [15,16]. Patients with multiple silent LIs showed a poorer cognitive performance related to short delayed verbal memory and frontal functions than patients with a single LI. For this reason, some studies have suggested that this type of silent lacunes had a significant individual contribution to the risk of cognitive impairment and motor speed and executive functions, while global cognitive functions and memory functions remained unaffected [17,18]. On the other hand, cognitive impairment associated with vascular disease frequently does not fulfill the traditional criteria for degenerative dementia, because the criteria of Alzheimer’s disease requires the presence of prominent memory impairment, which in contrast is not the main sign of vascular cognitive impairment. Patients with vascular dementia show a better long-term memory and greater deficits in frontal executive functioning than patients with Alzheimer’s disease [18]. The neuropsychological assessment reveals planning, organization and abstraction impairment, as well as deficits in category fluency initiation, reasoning, mental flexibility, sequencing, fine motor performance, or attentional allocation source [19-21].

It may be argued that multiple silent LIs may be associated with a higher risk of cognitive impairment in the mid- and long-term [22]. However, further studies are needed to assess differences in prognosis regarding cognitive function in patients with single LI versus patients with multiple silent LIs. Cognitive stimulation and physical activities could be recommended to improve affected cognitive functions. In the LADIS (Leukoaraiosis and Disability) study, physical activity reduced the risk of cognitive impairment, mainly vascular dementia, in older people living independently [23].

A limitation of the study is the fact that leukoaraiosis was assessed qualitatively as presence versus absence and not quantified using a quantitative measure (e.g. Scheltens scale, Wahlund scale, Fazekas scale). In a community sample of asymptomatic participants aged 50 to 65 years, it has been shown that only deep white matter hyperintensities, not periventricular hyperintensities were related to diminished cognitive function in middle-aged individuals [24]. The LADIS study [25] has shown that white matter hyperintensity volume was the strongest predictor of cognitive decline in cerebral small vessel disease, but also incident lacunes on MRI parallel a steeper rate of decline in executive functions and psychomotor speed. Accordingly, in addition to white matter lesions, lacunes determine longitudinal cognitive impairment in small vessel disease. Although the individual contribution of lacunes on cognition was modest, they cannot be considered benign findings, but indicate a risk of progressive cognitive impairment. In the present study carried out in patients with first-ever lacunar stroke and without cognitive impairment (MMSE mean score of 27.8 and 28 in the groups of single LI and multiple silent LIs, respectively), early neuropsychological alterations could be initially related to lacunes. A subsequent increase in leukoaraiosis may cause more significant neuropsychological alterations of the type of subcortical cognitive impairment. Subcortical LIs have been identified as the cause of mild cortical atrophy [26] and, although this was not analyzed in the present study, may also influence on mild incipient neuropsychological alterations observed in our patients.

The fact that some patients were included in a clinical trial (SPS3 randomized trial) of secondary prevention of cerebral ischemia (aspirin 325 mg/day versus aspirin 325 mg/day plus clopidogrel 75 mg/day) may be view as a limitation of the study. However, participation in the trial did not interfere with radioimaging and neuropsychological studies of the patients because all those included in the present study presented a first-ever lacunar stroke.

## Conclusions

The presence of multiple silent LIs documented on brain MRI scans in patients with first-ever lacunar stroke 1 month after the acute cerebrovascular event was associated with mild neuropsychological abnormalities, particularly in the performance of executive functions (semantic fluency) and short delayed verbal memory. It has been shown that silent lacunes themselves exert an independent effect on cognitive function [27]. In the initial stages of small vessel disease, mild neuropsychological abnormalities appear to be related to lacunes rather than to leukoaraiosis or perivascular hyperintensities of vascular cause.

## Abbreviations

CI: Confidence interval; CVLT: California verbal learning test; LI: Lacunar infarction; MMSE: Mini mental state examination; MRI: Magnetic resonance imaging; OR: Odds ratio; SD: Standard deviation; SVD: Subcortical vascular dementia; WAIS-III: Wechsler adult intelligence scale version III.

## Competing interests

The authors declare that they have no competing interests.

## Authors’ contributions

LB was the principal investigator, designed the study, collected the data, did the neuropsychological assessments, contributed to analyze the data, interpreted the results and wrote the paper. AA diagnosed and took care of the patients, participated in the study design, analysis and interpretation of data and wrote the part of the paper related to the neurological issues. He is also the corresponding author. DC participated in the collection of data, medical care of the patients and review of the manuscript for intellectual content. MGO contributed to write the paper, edited the manuscript and provide editorial assistance, including the selection of the journal. JCSM and OP participated in the collection of data, medical care of the patients and review of the manuscript for intellectual content. All authors have read and approved the final draft.

## Pre-publication history

The pre-publication history for this paper can be accessed here:

http://www.biomedcentral.com/1471-2377/13/203/prepub
